# Bone marrow mesenchymal stem cells combine with Treated dentin matrix to build biological root

**DOI:** 10.1038/srep44635

**Published:** 2017-04-12

**Authors:** Shiwei Luo, Fei Pei, Wen Zhang, Weihua Guo, Rui Li, Wei He, Weidong Tian

**Affiliations:** 1 Department of Oral and Maxillofacial Surgery, The First Affiliated Hospital of Zhengzhou University, Zhengzhou, China; 2 National Engineering Laboratory for Oral Regenerative Medicine, West China Hospital of Stomatology, Sichuan University, Chengdu, China; 3 State Key Laboratory of Oral Diseases, West China Hospital of Stomatology, Sichuan University, Chengdu, China; 4 Department of Pedodontics, West China School of Stomatology, Sichuan University, Chengdu, China; 5 Department of Oral and Maxillofacial Surgery, West China School of Stomatology, Sichuan University, Chengdu, China

## Abstract

Treated dentin matrix (TDM) as a kind of scaffolding material has been proved odontogenic induction ability on dental-derived stem cells. Given the limited resources of dental stem cells, it is necessary to seek new seed cell which easily obtained. Jaw bone marrow mesenchymal stem cell (JBMMSC) as non-dental-derived stem cell relates to the development of teeth and jaws which suggest us JBMMSCs could act as a new seed cell for tooth tissue engineering. To assess the odontogenic and osteogenic potential of JBMMSCs, cells were induced by TDM extraction *in vitro* and combined with TDM *in vivo*. Results were analyzed by PCR, Western Blotting and histology. PCR and Western Blotting showed odontogenic and osteogenic makers were significantly enhanced in varying degrees after induced by TDM extraction *in vitro. In vivo*, JBMMSCs expressed both odontogenic and osteogenic-related protein, and the latter showed stronger positive expression. Furthermore, histological examination of the harvested grafts was observed the formation of bone-like tissue. Therefore, osteogenic differentiation ability of JBMMSCs were enhanced significantly after being inducted by TDM which illustrates that non-odontogenic derived stem cells are still promising seed cells in tooth root tissue engineering.

Tooth loss due to periodontal diseases, dental caries, mechanical trauma, endodontic complications and other diseases is the most common organ failure^1^. While it is not usually a life-threatening condition, tooth loss greatly impacts the quality of life. Conventionally, lost teeth are replaced with artificial dentures, such as removable partial dentures, fixed partial dentures and dental implants. However, the high failure rate of artificial dentures has been attributed to the development of caries, periodontal diseases and endodontic pathology^2^.

Stem-cell based tissue engineering, using isolated cell suspensions and biological scaffolds, has been successful in reconstructing several tissues, such as bladder, airway, blood vessels and bone. Advancement in tissue engineering has enabled us to contemplate a new and promising strategy for hard tissue repair, particularly for tooth tissue reconstruction with normal physiological function^3^,^4^.

Dental tissue engineering involves the harvest of stem cells from the patient, then inducing these stem cells towards a relevant lineage on a suitable scaffolding material, and finally reintroducing the cell-scaffold construct to the patient^3^. Stem cells derived from dental tissues are ideal seeding cells for the reconstruction of dental tissue^5^,^6^. However, the source of dental stem cells is limited, especially edentulous jaw patients^1^. Besides, obtaining postnatal dental stem cells in the clinic is difficult, maybe even impossible. And immunosuppression represents a crucial barrier to the transplantation of both heterogeneous and homogenous cells^7^. It is a key challenge to finding suitable seeding cells. Adult bone marrow mesenchymal stem cells (BMMSCs) not only could be obtained easily but also have the ability to differentiate into multilineage cells. These cells could be induced to osteoblasts, adipocytes, chondrocytes, and even muscle and neural tissues^8^,^9^. In general, dental stem cells possess stronger odontogenic capability than non-dental stem cells. After dental follicle cells (DFCs) combined with TDM to be implanted into alveolar fossa of adult rats for four weeks, root-like tissues stained positive for markers of dental pulp and periodontal tissues were regenerated^4^. Periodontal ligament stem cells (PDLSC) mixed with hydroxyapatite/tricalcium phosphate ceramic particles could generate a cementum/PDL-like structure and contribute to periodontal tissue repair when transplanted into nude mice subcutaneously, while bone marrow structures were generated by bone marrow stromal stem cells (BMSSCs)^10^ which is another term of bone marrow mesenchymal stem cells. After cultured for 14 days in the renal capsules of rat hosts, recombined dental pulp stem cells (DPSC)/apical bud cells (ABC) pellets formed typical tooth-shaped tissues with balanced amelogenesis and dentinogenesis, whereas BMSSC/ABC recombinants developed into atypical dentin–pulp complexes without enamel formation^11^. On the other hand, with appropriate inductive microenvironment (such as growth factors and morphogens), BMMSCs could also differentiate into odontoblasts and resulted in the formation of tooth structures when transplanted into nude mice^12^. Above experiments remind us that maybe BMMSCs could play as the seed cells to build biological root. Furthermore, the use of autogenous BMMSCs for tissue regeneration prevents immunologic rejection. Thus, the study of replacing dental mesenchyme by BMMSCs is significant for tooth regeneration research.

In addition to seeding cells, the identification and development of suitable scaffolding materials capable of regenerating tooth structures, such as dentin, are essential aspects of tooth tissue engineering^13^,^14^. To date, several scaffold materials have been used in tooth tissue engineering. However, most of them result in incomplete tooth tissue regeneration because of lacking the property of inducing odontogenesis^15^,^16^. A suitable scaffolding material is non-cytotoxic, bioactive, and capable of providing a three-dimensional microenvironment supporting cell growth, differentiation, and cellular organization into a tissue structure^3^. As a major component of the tooth, dentin matrix contains a variety of bioactive molecules such as growth factors, bioactive molecules, signaling molecules, and transcription factors^17^. Recently, we took advantage of dentin matrix and fabricated a natural scaffold, treated dentin matrix (TDM), for tooth tissue engineering^3^. TDM could induce odontogenesis in the dental follicle cells with good biocompatibility and bioactivity. Because of a large amount of odontogenic bioactive proteins and factors contained in TDM^4^,^18^, we propose the hypothesis that TDM may be a suitable scaffold and an inductive microenvironment for the construction of the dental tissues. With the odontogenic induction of TDM, JBMMSCs may differentiate into dental tissues. Furthermore, numerous proteins and growth factors could be secreted through JBMMSCs differentiation, which participates in dentinogenesis during tooth development^19^,^20^,^21^,^22^. Besides, how to control the shape of tissue engineering tooth is poorly understood^23^. As natural materials obtained from the human tooth, TDM can be easily fabricated into any shape.

Although we have regenerated complete dentin tissue with TDM and dental follicle cells, some problems remain to be solved. Assessment of whether TDM has the capacity to induce non-dental cells, such as bone marrow stem cells, into an odontogenic specialty are of interest. Therefore, this study is a continuation of our previous study. In this study, we investigated the odontogenic inductive capacity of TDM on JBMMSCs *in vitro*. Ultimately, this work was aimed to assess whether TDM can provide a suitable scaffold and inductive microenvironment for JBMMSCs to regenerate dental tissues *in vivo.*

## Results

### Morphology and Immunofluorescent microscopy of JBMMSCs

**Figure 1:**
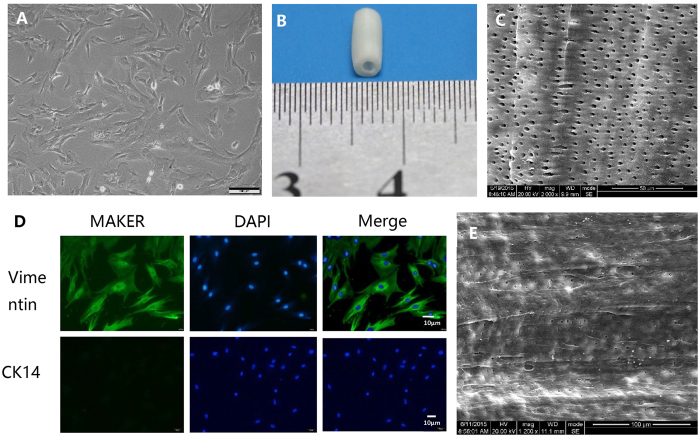
Identification and characteristics of JBMMSCs and TDM. Second-passage cells were typical spindle or triangle shape morphology (**A**) and positive for Vimentin but negative for CK14 in immunofluorescence staining (**D**). TDM (**B**) as scaffold was fabricated from human healthy teeth. SEM (**C**) illustrates that dentinal tubules exposed sufficiently and loosened fiber bundles of intertubular and peritubular dentin. (**E**) JBMMSCs were planted on the surface of TDM. Scale bars = 100 μm, 10 μm for (**A**,**D)** respectively.

Human JBMMSCs are mesenchymal cells with a typical spindle or triangle shape morphology (Fig. 1A). After two passages, the typically fibroblastic JBMMSCs were purified *in vitro*. JBMMSCs were positive for vimentin, which are proteins characteristic of mesenchymal stem cells, but were negative for the marker of epithelial cells, CK-14 (Fig. 1D).

### Surface molecule characterization of JBMMSCs

FCM analysis about surface molecule revealed that JBMMSCs were positive for mesenchymal stem cell markers CD29, CD73, CD90, CD105, CD146 while negative for platelet endothelial cell adhesion molecule-1 CD31 and hematopietic stem cell marker CD 34 (Fig. 1C) which were in accord with mesenchymal stem cells.

### Multipotential differentiation of JBMMSCs

**Figure 2:**
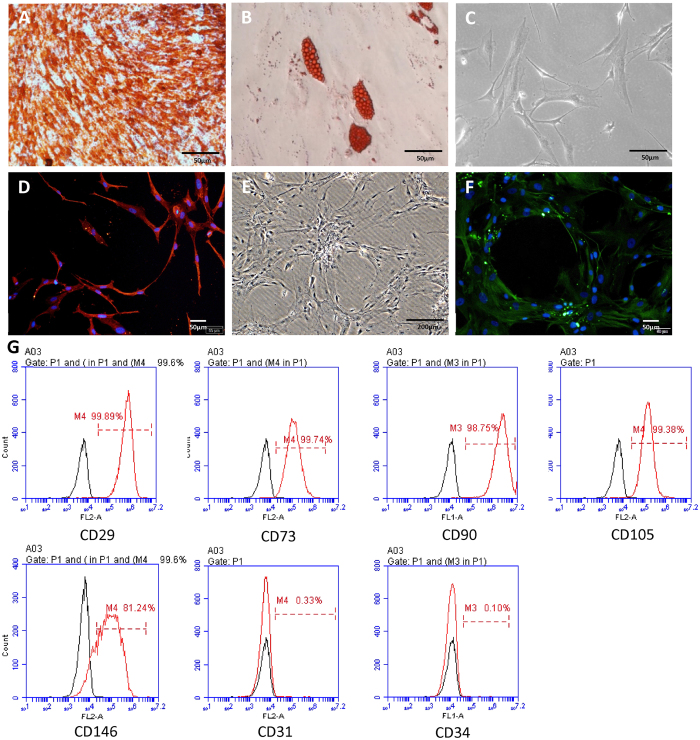
Characteristics of JBMMSCs. Panel shows lipid clusters (**A**) mineralized nodules (**B**) were observed respectively when JBMMSCs were cultured in osteogenic, adipogenic inductive medium for 2 and 4 week. JBMMSCs were transformed into neuron-like cells (**C**) and vascular endothelial-like cells (**E**) in neurogenic and vascular inductive medium. Moreover, these neuron-like cells stained positively for nestin (**D**) and vascular endothelial-like cells were positive for CD31 (**F**) in immunofluorescence. (**G**) Flow cytometry indicated that JBMMSs were positive for mesenchymal stem cell surface marker CD29, CD73, CD90, CD105, CD146 while negative for platelet endothelial cell.adhesion molecule-1 CD31 and hematopietic stem cell marker CD 34. Scale bars = 50 μm for (**A**–**D**,**F**), Scale bars = 200 μm for (**E**).

After induction in osteogenic medium for 2 weeks, JBMMSCs formed a large number of mineralized nodules that were observed by alizarin red staining (Fig. 2A). Following being cultured in adipogenesis inducing medium for 4 weeks, lipid droplets were observed when stained with oil red (Fig. 2B). JBMMSCs were transformed into neuron-like cells (Fig. 2C) which were acerose and maintained contact with other cells through synapse-like structure in neurogenic inductive medium. Cells cultured in vascular inductive medium became vascular endothelial-like cells in circle arrangement (Fig. 2E). Moreover, these neuron-like cells stained positively for nestin (Fig. 2D) and vascular endothelial-like cells were positive for CD31 (Fig. 2F) in immunofluorescence. The above results demonstrated that thes cells had potential for differentiating to osteoblast, lipoblast, nerve-like cell and vascular endothelial-like cells with the multiple differentiation potential.

### Fabrication of human TDM

Human TDM was fabricated using the method described in our previous study^3^. It was produced to the dimensions of 1.0–1.5 cm in length and 1.0 mm in thickness. SEM examination demonstrated that the dentinal tubules were sufficiently exposed and fiber bundles of intertubular and peritubular dentin were loosened after being treated with EDTA (Fig. 1B,C).

### Real-time PCR analysis

**Figure 3:**
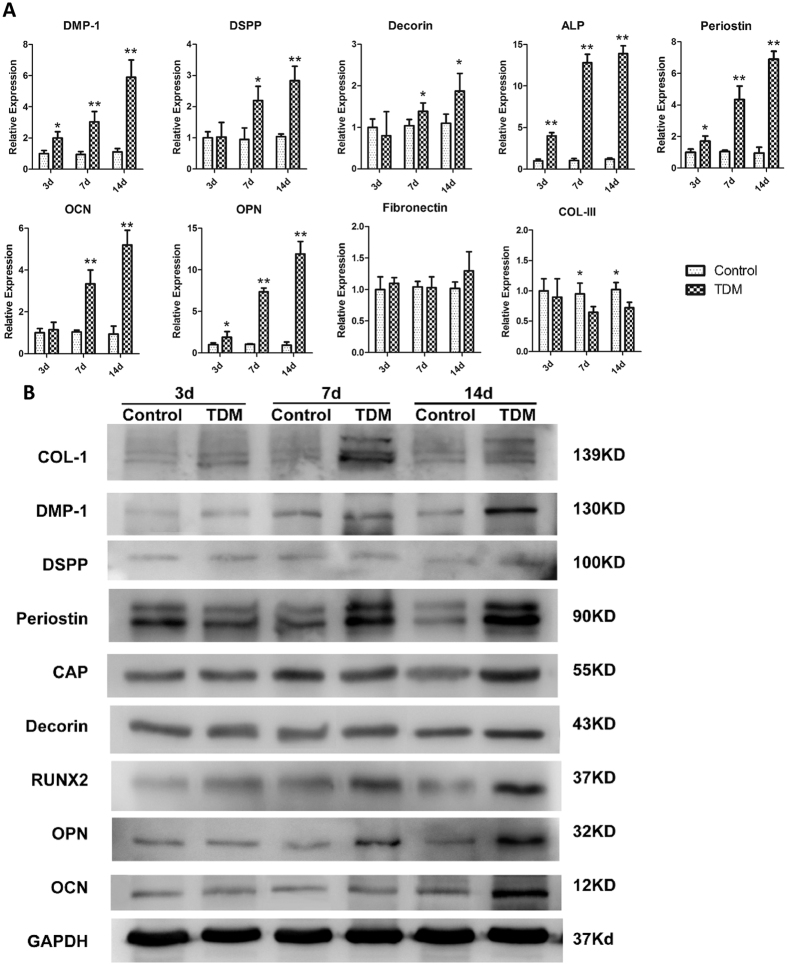
Real-time qPCR (**A**) and Western Blotting (**B**) were performed to assess the effect of the liquid extraction of TDM on the JBMMSCs at 3 d, 7 d, 14 d respectively. Overall, (**A**) TDM up-regulated the expression of odontogensis markers DSPP and DMP-1, osteogenesis marker Periostin and OCN, early stage osteogenic differentiation marker ALP and dentin noncollagen protein Decorin than control group but kept the expression level of Fibronectin. Meanwhile, the expression of COL-III was down-regulated after inducement (**B**) Western Blotting showed JBMMSCs expressed the odontogenesis related protein DMP1 and osteogenesis related protein OPN, OCN, CAP, Periostin, and RUNX2 stronger in varying degrees than control group at later time points. Interestingly, DSPP and Decorin maintained a stable expression as well as the control group. (*p < 0.05, **p < 0.01).

Real-time PCR was performed to assess the effect of the liquid extraction of TDM on the JBMMSCs. Odontogenic-related and osteogenic-related genes like DMP-1, ALP, Periostin, OPN not only showed higher expression than control group at all time points but also expressed stronger and stronger as time went on. Expression of DSPP, Decorin and OCN kept stable with control group at 3 d and began to rise at later time points. However, there was no significant difference between experiment group and control group about the expression of fibronectin and COL-III which were not the mineralization related marker (Fig. 3A).

### Western Blotting

Western Blotting was also used to assess the effect of the liquid extraction of TDM on the JBMMSCs. JBMMSCs expressed the odontogenesis-related protein DMP-1 and osteogenesis-related protein Periostin, CAP, OPN, OCN, stronger in varying degrees than the control group just at later time points which was similar to the trend of gene expression. Expression of COL-1 and RUNX2 showed up-regulation in the whole inducing process. Interestingly, DSPP and Decorin maintained a stable expression as well as the control group (Fig. 3B). Statistical analysis of gray values was showed in Supplementary Fig. S2.

### Tissue regeneration *in vivo* using TDM and JBMMSCs

**Figure 4:**
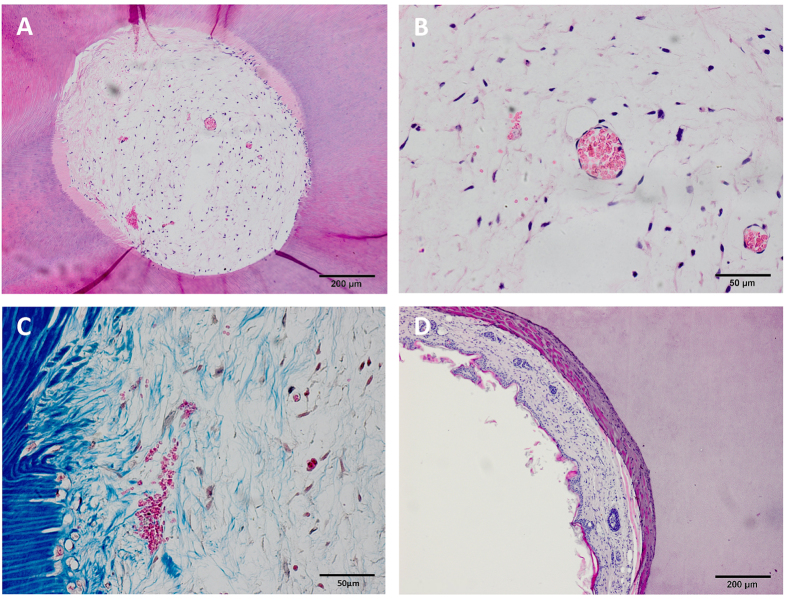
H&E staining showed planted cells had ideal adherence and proliferation on TDM (**A**). JBMMSCs peformed connective tissue-like construction with new vessel generation (**B**). However expected regenerated dentin such as dentinal tubules, predentin, was not observed which is same in control group (single TDM) (**D**). Masson stain (**C**) showed the fibrous tissue performed. Scale bars = 200 μm for (**A**,**D**). Scale bars = 50 μm for (**B**,**C**).

**Figure 5:**
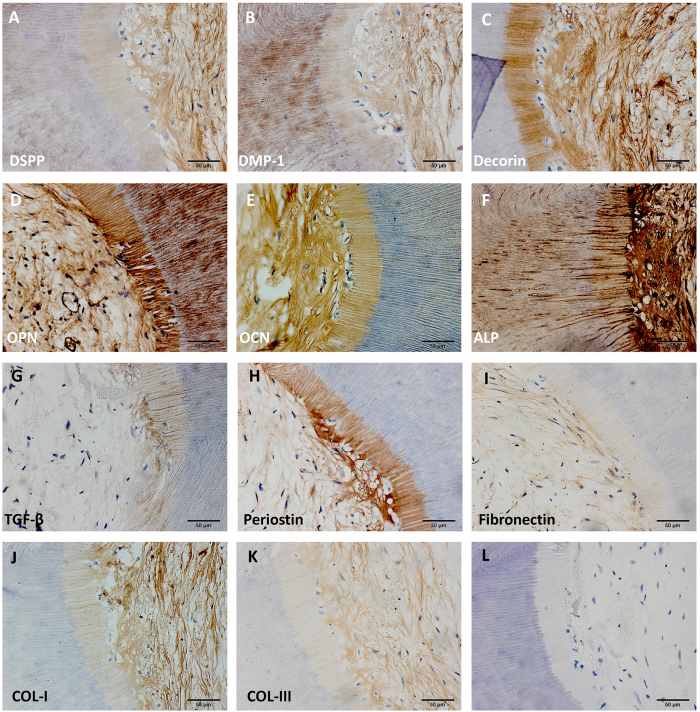
Immunohistochemistry was used to evaluate the expression of DSPP, DMP-1, OCN, OPN, Decorin, TGF-β, COL-I, COL-III, Periostin, Fibronectin and ALP to explored the differentiation status of JBMMSCs. The result showed that JBMMSCs were strongly positive for protein osteogenic marker OPN (**D**) and ALP (**F**) not only in the cells but also in demineralized layer and dentin. Positive expression of OCN (**E**), Decorin (**C**) and Periostin (**H**) were also observed in cells and demineralized layer. Odontogenic markers DSPP (**A**), DMP-1 (**B**) and normal dental pulp collagen fibrils components COL-I (**J**), COL-III (**K**) showed weakly positive expression. However, most cells were stained negatively for TGF-β and Fibronectin Scale bars = 50 μm.

**Figure 6:**
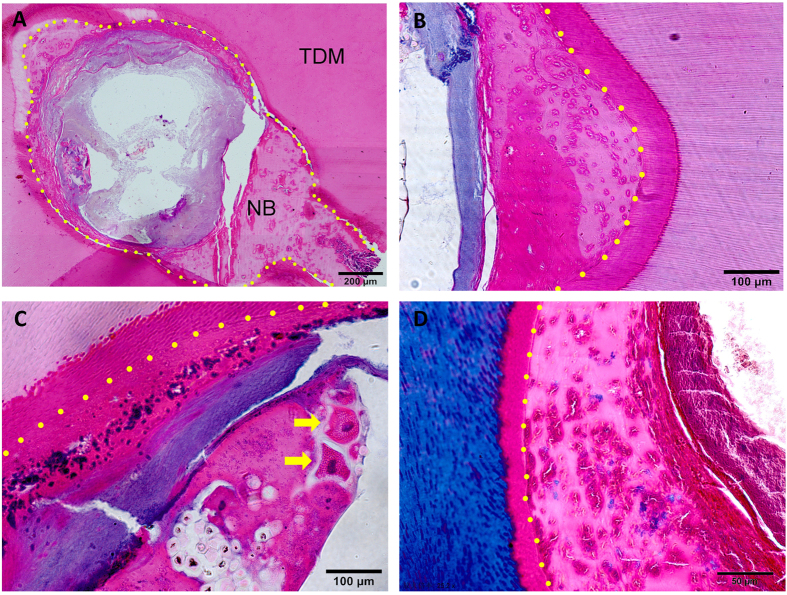
Formation of new tissue. (**A**) The bone like tissue were formed on the pulp cavity surface of TDM. Many osseous lacunas spreaded over newly-formed bone matrix (**B**). We could also observe the osteoblast like cells at the edge of bone matrix (The yellow arrows had pointed out) (**C**). In Masson staining, newly-formed bone like tissue were stained red instead of blue suggested that the tissue contained less collagen components (**D**). (The yellow spots labeled the layer of newly-formed bone-like tissue, TDM: treated dentin matrix, NB: newly-formed bone-like tissue). Scale bars = 200 μm for A. Scale bars = 100 μm for (**B**,**C**). Scale bars = 50 μm for (**D**).

All experimental animals recovered quickly, showing no inflammatory response after implant surgery indicating good biocompatibility of human TDM. H&E staining showed planted cells had ideal adherence and proliferation on TDM (Fig. 4A). Besides we observed that there was a lighter stained layer inside TDM. We thought that structure was demineralized dentin due to demineralization in fabricating. JBMMSCs performed connective tissue-like construction with new vessel generation (Fig. 4B). However expected new tissue was not observed in control group (single TDM) (Fig. 4D). Then we used immunohistochemistry to evaluate the expression of DSPP, DMP-1, OCN, OPN, Decorin, TGF-β, COL-I, COL-III, Periostin, Fibronectin and ALP to explored the differentiation status of JBMMSCs. The result showed that JBMMSCs were strongly positive for protein osteogenic marker OPN (Fig. 5D) and ALP (Fig. 5F) not only in the cells but also in dentin and demineralized dentin layer. Positive expression of OCN (Fig. 5E), Decorin (Fig. 5C) and Periostin (Fig. 5H) was also observed in cells and demineralized layer. Odontogenic markers DSPP (Fig. 5A), DMP-1 (Fig. 5B) and normal dental pulp collagen fibrils components COL-I (Fig. 5J), COL-III (Fig. 5K) showed weakly positive expression. However, most cells were stained negatively for TGF-β (Fig. 5G) and Fibronectin (Fig. 5I). It is interesting that we found the bone like tissue which formed on the pulp cavity surface of TDM in some samples (Fig. 6A). Many osseous lacunas spreaded over neoformative bone matrix (Fig. 6B). We could also observe the osteoblast-like cells at the edge of bone matrix (Fig. 6C). In Masson staining, neo-formative bone-like tissue was stained red instead of blue suggested that the tissue contained fewer collagen components (Fig. 6D).

## Discussion

Many materials have been adopted as scaffolds for dentin regeneration^24^,^25^,^26^. However, these materials can hardly contribute to prefabricated-shaped and complete dentin regeneration. The reason may be that while these materials support cell growth and mineralization, they are not capable of inducing differentiation towards an odontogenic specialization. As the main component of tooth, dentin is less mineralized and more elastic than enamel. It is reported that the organic matrix of dentin contain approximately 30 volume percent of collagen, noncollagenous proteins (NCPs), and growth factors, and many of these proteins and factors have been shown to be important in dentin development, mineralization, and regeneration^17^,^27^,^28^. Besides, it has been reported that a three-dimensional (3D) microenvironment with specific properties that could promote *in vitro* proliferation of chondrocytes, hepatocytes, endothelial cells, osteoblasts, neuronal cells, and stem cells^29^. As a natural decellularization matrix scaffold, TDM provided a 3D odontogenic inductive microenvironment. The odontogenic potential of dentin matrix on dental stem cells has been confirmed, whether TDM could also induce non-dental stem cells differentiate into an odontogenic specialization is not realized.

The present results showed that BMMSCs stemmed from human jaw were generally typical spindle or triangle shape. Importantly, cells stained positive for vimentin but negative for CK14. As positive staining for vimentin and CK14 is indicative of mesenchymal and epithelial cells, respectively, this result indicated that these cells were mesenchymal but not contaminated by epithelial cells. FCM showed that cell surface molecules were positive for mesenchymal stem cell surface marker like CD29, CD73, CD90, CD105, CD146 but negative for hemopoietic stem cell marker CD31 and CD34 which belongs to platelet endothelial cell adhesion molecules. Furthermore, these cells had potential for differentiating to osteoblast, lipoblast, nerve-like cell and vascular endothelial-like cells which illustrated they were certainly the stem cells. These results proved that the cells from jaw marrow were indeed BMMSCs.

TDM as scaffold was fabricated from human healthy teeth with exposed dentinal tubules which. JBMMSCs could adhere to surface of framework due to good biocompatibility of TDM. In our previous study, we found TDM expressed COL-I, TGF-β1, decorin, biglycan, DMP-1, and DSPP^3^. In this study, we detected not only the odontogenic markers above but also some other markers such as OCN, OPN, Fibronectin.

As the most abundant protein, COL-I forms a visible network around the dentinal tubules in the peritubular region and in the intertubular predentin region, with multiple spherical mineral foci, which are distinctive features of dentin mineralization^30^. COL-1 is secreted by odontoblasts into predentin and then recruited into the mineralization front as bundles of collagen fibrils, thus forming mineralized. However, biglycan and decorin are considered to interact with COL-I fibrils in dentin mineralization^31^,^32^. By contrast, the content of COL-III is far less than that of COL-I. COL-III could be detected in dental pulp and periodontium and it plays a role in the development of trabecular bone through its effects on osteoblast differentiation^33^. TGF-β1 appeared in tooth morphogenesis and odontoblast differentiation during embryogenesis and could also be detected immunohistochemically in mature human teeth^34^,^35^. During tooth development, TGF-β expressed with a high level, which was considered to relate to the differential activity on the cells at distinct stages of development^36^,^37^. TGF-β1 could lead to the sequestration of these growth factors within the dentin matrix that could be released as a result of matrix changes associated with caries or trauma^38^. Other studies have also demonstrated that application of TGF-β1 could stimulate reparative dentin formation^39^,^40^. These data implicated TGF-β1 played a key role in the dentin tissue regeneration. As a primary component of noncollagenous proteins, DMP-1 and DSPP were also detected. DMP-1 is a multifunctional protein involved in the mineralization of bones and dentin^41^. DMP-1 can induce organization of apatitic crystalites into bundles with their c-axes co-aligned with the axis of the bundle, which is a hallmark of the collagenous mineralized tissues such as bone and dentin^42^. DSPP is also an important nucleator for dentin mineralization and proper dentin formation^28^,^43^. DSPP is expressed and secreted by odontoblasts, the cells that make tooth dentin and that also maintain cell processes extending into the mineralized tissue. Some studies suggest that a deficiency in DSPP is a causative factor in dentinogenesis imperfecta (DI)^43^. DSPP and DMP-1 expression was therefore up-regulated perhaps because TDM could lead JBMMSCs to osteogenic differentiation. Besides, biglycan and decorin were also expressed in a relatively high level of concentration, which was widely distributed in mammalian tissues, including mineralized tissues such as bone and teeth^32^,^44^. The ability of biglycan and decorin to bind to and inhibit the activity of TGF-β in previous studies makes these two proteoglycans likely candidates for the sequestration of TGF-β reservoirs in the dentin matrix^45^,^46^. In addition to modifying the extracellular environment, interactions of biglycan and decorin with cells, and with growth factors such as TGF-β1 affect the proliferation of cells^47^. Furthermore, increased levels of biglycan or decorin may be the cause or consequence of hypomineralization and widened predentin in Dspp−/− mice^48^. Both biglycan and decorin single gene knockout mice exhibited altered numbers of collagen fibrils in the molar predentin at a younger age^49^. Cementum attachment protein (CAP) is considered as cementum specific protein because of significantly higher expression in cementum than bone tissue. In addition, CAP is usually regarded as marker protein of cementoblast precursors with ability to promot proliferation and differentiation of periodontal ligament cells^50^. Runx2 as an important transcription factor has profound effects on osteoblast differentiation in the bone microenvironment. Runx2 target genes include regulators of cell growth control, components of the bone extracellular matrix, angiogenesis, and signaling proteins for development of the osteoblast phenotype and bone turnover^51^. Alkaline phosphatase (ALP) as a mineralization-related marker could participate in the differentiation of osteoblasts. Expression of ALP would become stronger following the increase in the degree of osteogenic differentiation which was similar to RUNX2. OCN secreted by osteoblasts, odontoblasts, and cementoblasts is a major noncollagenous protein found in bone and dentin and could restrict the mineralization of bone and tooth tissue, including^52^, and is regarded as a terminal symbol in hard tissue regeneration^53^. OPN is a multifunctional phosphorylated glycoprotein highly expressed in the hard tissue like bone, tooth. And as a kind of effective inhibitor of hydroxyapatite formation^54^, it could regulate the development of dentin. Periostin expressed in ameloblast, subodontoblast, and odontoblast cells. It plays a novel direct role in controlling postnatal tooth formation, which is required for the integrity of both enamel and dentin^55^. Periostin is the cell adhesion molecule of osteoblasts and their precursor cells. Previous research has shown that periostin located between the cytoplasmic processes of periodontal fibroblasts and cementoblasts and the adjacent collagen fibrils, and bear the mechanical stress in the process of tooth eruption and occlusion^56^.

In summary, these proteins and factors not only play important roles in inducing JBMMSCs proliferation and differentiation into odontoblasts but also form a network to form dentin tissues and control mineralization during dentin regeneration and development.

From the results of real-time PCR and Western Blotting, we observed that mineralization-related markers DMP-1, ALP, RUNX2, OCN, CAP, Periostin and COL-I were high-expression after induction by TDM, while the COL-III and Fibronectin were not significantly up-regulated after induction by TDM. This indicated TDM has the ability for odontogenic and osteogenic induction which may depend on the release of active factors due to exposure of dentin tubules after treated. And it is worth noting that osteogenesis-related makers such as OPN, RUNX2 possessed stronger expression compare with odontogenesis-related markers such as DSPP, Decorin. This suggested that JBMMSCs as a kind of non-dental stem cells may be inclined to differentiate in the direction of osteogenesis under the influence of the active factors from TDM. In order to further verify the validity of the odontogenic functions of JBMMSCs and TDM *in vivo*, TDM scaffolds seeded with JBMMSCs were implanted into mice at subcutaneous pocket with no mineralization capacity. JBMMSCs has an ideal attachment and proliferation on TDM, which was demonstrated by SEM (Fig. 1E). These represented the superior biocompatibility and bioactivity of TDM. With the induction of growth factors secreted by TDM, JBMMSCs have a greater mobility. Cell cytotoxicity test indicated good biocompatibility of TDM in our previous study^9^.

At first, we detected expression of human-mitochondria (Does not react with other species) in harvest tissue with immunohistochemistry to prove that these cells came from implanted human cells. Result showed the cells within TDM stained positive for human-mitochondria (Supplementary Fig. S1) which illustrated they were human cells while not the mouse cells from the host. From HE staining, we could observe the pulp-like tissue and new blood vessel generated (Fig. 4B), new fibrous tissue were also observed by Masson stain (Fig. 4C), but there was no obvious dentin or predentin formation. Immunohistochemistry showed JBMMSCs gained a greater degree in osteogenic differentiation than odontogenic differentiation (Fig. 5A–L). Moreover, JBMMSCs were capable of promoting mineralization of demineralized dentin layer (Fig. 3A–F,H). Therefore JBMMSC tended to osteogenic differentiating instead of differentiating to dentin or dental pulp tissue with the effect of TDM.

Our results indicated that JBMMSCs were capable of differentiating into multiple cell lineages when grown under defined conditions *in vitro* (Fig. 2A,B), which was in accordance with previous studies^57^,^58^. A previous study showed that human dental pulp stem cells (DPSCs) and bone marrow stem cells had similar gene expression levels of more than 4,000 known human genes^59^. JBMMSCs share numerous characteristics with dental stem cells and are both able to form bone-like or tooth-like structures. In this study, we found that the odontogenic potential of JBMMSCs was enhanced by TDM. The reasons may be that a lot of odontogenic proteins and molecular signals regulating the fate of tooth development, such as Notch signals, TGFs (transforming growth factors), BMPs (bone morphogenetic proteins) and FGFs, are expressed permanently in TDM. However, we found that JBMMSC showed higher level osteogenic potential according to results of PCR and immunochemistry. JBMMSCs display a lower odontogenic potential than dental stem cells, indicating that MSC from different embryonic origins are not equivalent^11^. Furthermore, the formation of bone-like tissue also indicated the osteogenic potential of JBMMSC and TDM could induce the osteogenic differentiation of JBMMSC. On the other side, as we know, JBMMSCs could be induced to osteoblasts due to favorable microenvironments, and also osteoclasts due to unfavorable microenvironments. So it is necessary to avoid internal absorption process of TDM framework *in vivo*. There exists a kind of coupling relationship between osteoblast and osteoclast so that bone mass is maintained locally by the balance between osteoclastic bone resorption and osteoblastic bone formation^60^. So osteoclasts are inevitable when TDM with JBMMSCs is implanted in alveolar bone. TDM could induce mineralization by releasing bioactive factors^3^. The existence of osteoclasts would be conducive to bone remodeling instead of bone resorption if there is no infection or immunologic rejection after implantation. Furthermore, our previous studies showed that there was no bone resorption but tissue regeneration observed after implanted TDM combined with dental follicle cells into alveolar bone^4^,^18^,^61^. We, therefore, believe that without infection or immunologic rejection, severe bone resorption will not occur when implantation in alveolar bone.

As for no dentin, predentin was observed, in our opinion, the formation of complete dentin, predentin, pulp nerve structures is a complex process which requires regulation of many genes and proteins. While JBMMSCs as a non-dental stem cells combine with TDM generate bone-like tissue subcutaneously in nude mice makes synosteosis possible between TDM and alveolar bone when being transplanted into the jaws in the future.

## Conclusion

Here we have described the odontogenic potential of JBMMSCs induced by TDM both *in vitro* and *in vivo*. Our results confirmed the good biocompatibility and odontogenic microenvironment of TDM on non-dental derived stem cells. We found JBMMSCs could express odontogenic and osteogenic markers which demonstrated that JBMMSCs have odontogenic potential, the ability to differentiate into odontoblast-like cells and contribute to odontogenesis. Although JBMMSC possesses stronger ability in promoting mineralization and osteogenic differentiation, even could form bone-like tissue with TDM inductive effect, it is still prospective seed cell in building biological root for tooth regeneration.

## Materials and Methods

All experiments were conducted in accordance with the ethical protocol approved by the local ethical committee of the Sichuan University. All the methods were carried out in accordance with the approved guidelines. In addition, for investigations involving human subjects, informed consent has been obtained from the participants involved.

### JBMMSCs isolation and culture

Human JBMMSCs were isolated from mandibular bone marrow and the cancellous bone fragment of the consenting donor who received orthognathic surgery due to malocclusion. The donor was examined to exclude hematopoietic neoplasms and the samples were found to be histologically normal. We applied the whole bone marrow culture method to culture JBMMSCs and purified the cells through differential adherent time. Depurated cells were cultured in the medium consisting of Dulbecco’s modified Eagle’s medium (DMEM, Hyclone, USA), 10% fetal bovine serum (Hyclone, USA), 100 U/mL penicillin, and 100 μg/mL streptomycin (all Sigma, USA). Cells were seeded at a density of 2 × 10^5^ cells/cm^2^. After 72 h, non-adherent cells and debris were washed out by the first media exchange. Then, cells were incubated in DMEM supplemented with 10% fetal bovine serum in a humidified atmosphere at 37 °C and 5% CO_2_. Cell culture medium was changed every 2 d and cells from passages 2–4 were used for the experiments. After about 7 d, the JBMMSCs were observed under a phase-contrast inverted microscope (Nikon, Japan).

### Immunofluorescent microscopy of JBMMSCs

One day prior to staining, JBMMSCs were released and seeded onto 0.8 cm × 0.8 cm coverslips in a 24-well plate for further culture. JBMMSCs were fixed with 4% polyoxymethylene for 30 min. Subsequent steps were performed according to the manufacturer’s recommendations. Antibodies used in immunofluorescent staining included: vimentin and CK-14. All antibodies were from Abcam, USA. All samples were examined under a fluorescence microscope (Leica Optical, Germany).

### Surface molecule characterization and gene expression pattern of JBMMSCs

Approximately 5 × 10^5^ JBMMSCs were incubated with anti-CD105 (1:100; BD, CA, USA), anti-CD31 (1:100; BD, CA, USA), anti-CD34 (1:100; BD, CA, USA), anti-CD90 (1:100; BD, CA, USA), anti-CD73 (1:100; BD, CA, USA), anti-CD29 (1:100; Bio-Legend, CA, USA), anti-CD45 (1:100; BD, CA, USA), according to the manufacturers’ protocols. FITC-conjugated, isotype-matching immunoglobulins were used to determine non-specific staining. The secondary reagents included goat anti-mouse and goat anti-rat IgG-FITC (Santa Cruz). Cells were analyzed on an Accuri C6 Flow cytometer (Becton Dickinson, CA, USA), and the data were analyzed using CellQuest software.

### Multipotential differentiation of JBMMSCs

#### Osteogenic differentiation

A total of 1 × 10^5^ JBMMSCs were seeded into each well of a six-well plate. At 80% confluence, JBMMSCs were cultured in osteogenic medium containing 10% FBS, 5 mL glycerophosphate (Sigma, USA), 100 nM dexamethasone (Sigma, USA), and 50 mg/ml ascorbic acid (Sigma, USA) for 2 weeks. The control group was cultured in DMEM with 10% FBS. The medium was changed every two days. After 2 weeks, cells were washed twice in PBS after being fixed in 4% paraformaldehyde for 10 min and then incubated in 0.1% alizarin red solution (Sigma, USA) in Tris-HCl (pH 8.3) at 37 °C for 30 min. After being washed twice with PBS, cells were routinely observed and photographed under a light microscope (OLYMPUS, Japan).

#### Adipogenic differentiation

A total of 1 × 10^5^ JBMMSCs were seeded into each well of a six-well plate. At 80% confluence, JBMMSCCs were cultured in complete adipogenic medium consisted of DMEM supplemented with 10% FBS, 2 mM glutamine (Sigma, USA), 100 U/ml penicillin-streptomycin (Sigma, USA), 100 μM ascorbic acid (Sigma, USA), 0.5 mM methylisobutylxantine (Sigma, USA), 0.5 mM hydrocortisone (Sigma, USA) and 60 mM indomethacin (Sigma, USA). The control group was cultured in DMEM with 10% FBS. The medium was changed every 2 d. After 4 weeks, the cells growing under adipogenic conditions were washed twice with PBS and fixed in 70% ethanol for 10 min. Then the cells were incubated in 0.3% Oil Red O (Sigma, USA) solution for 15 min. After being washed three times with PBS, cells were routinely observed and photographed under a phase-contrast inverted microscope (OLYMPUS, Japan).

#### Neuronal differentiation

1 × 10^5^ JBMMSCs were seeded into wells of a six-well plate. At 80% confluence, JBMMSCs were cultured in neuronal induction medium DMEM-low glucose containing 2% DMSO, 20 μM butylated hydroxyanisole, 25 mM KCL, 2 mM valproic acid, 10 μM forskolin, 1 μM hydroxycortisone, 5 μg/mL insulin, and 2 mM L-glutamine. Detect the expression of neuronal marker-proteins after 2 days in culture. Induced JBMMSCs were washed twice with PBS and conventionally fixed with 4% polyoxymethylene for 10 min. Subsequent steps were performed according to the manufacturer’s recommended protocol. Primary antibody: monoclonal mouse to Nestin (1:200; Abcam, USA). PBS instead of the primary antibody was used as negative controls. The results were examined under a fluorescence microscope (OLYMPUS, Japan).

#### Vascular differentiation

1 × 10^5^ JBMMSCs were seeded into wells of a six-well plate. 80% confluent cells were cultured in vascular induction medium DMEM-low glucose containing 2%FBS and 50 ng/ml VEGF (PeproTech, USA) for 7 days. The medium was changed every 2 days. Induced JBMMSCs were washed twice with PBS and conventionally fixed with 4% polyoxymethylene for 10 min. Subsequent steps were performed according to the manufacturer’s recommended protocol. Antibody was rabbit anti CD31 (1:150, BioSS, USA). PBS instead of the primary antibody was used as negative controls. The results were examined under a fluorescence microscope (OLYMPUS, Japan).

### Fabrication of treated dentin matrix (TDM)

Human TDM was fabricated according to our previous method^3^. Briefly, premolar teeth that were removed for clinical reasons at the West China Hospital of Stomatology of Sichuan University were collected.

Periodontal tissues were completely scraped away with a curette. By grinding along the tooth profile, outer cementum and part of the dentin were removed. Dental pulp tissues and pre-dentin were also mechanically removed. The resulting human dentin matrix was soaked in deionized water for 5 h and mechanically cleaned for 20 min every hour using an ultrasonic cleaner. The deionized water was changed every hour. Human dentin matrices were then treated with 17% Ethylene Diamine Tetra-acetic Acid (EDTA, Sigma, USA) for 5 min, 10% EDTA for 5 min, 5% EDTA for 10 min. Human TDM were maintained in sterile phosphate buffered saline (PBS) with 100 units/ml penicillin (Hyclone, USA) and 100 μg/ml streptomycin (Hyclone, USA) for 72 h, then washed in sterile deionized water for 10 min in an ultrasonic cleaner, and finally stored in DMEM (Hyclone, USA) at 4 °C. Morphological observations of human TDM were observed by scanning electron microscope (SEM) (Inspect F, FEI, Netherlands).

### Effect of TDM on biological characteristics of JBMMSCs *in vitro*

To evaluate the effect of TDM on JBMMSCs, we first collected the liquid extract of the dentin matrix according to the protocol of the International Standardization Organization (ISO 10993). Briefly, scaffold samples of dentin matrix were pulverized using a cold mortar and pestle after freezing in liquid nitrogen. DMEM was added to the pulverized scaffold with a ratio of 20 g scaffold powder per 100 mL DMEM. Slurries were incubated for 2 d at 37 °C, and the samples were filtered using a 0.22 μm filter.

The JBMMSCs induced by TDM liquid extract were used as experimental groups (TDM liquid extract with 10% FBS). The JBMMSCs cultured in normal medium DMEM were used as control groups (DMEM with 10% FBS).

### Real-time PCR analysis

At 3, 7, 14 days, Real-time PCR was used to detect the differences in gene expression between JBMMSCs induced by TDM and JBMMSCs cultured in normal medium DMEM. RNA was extracted using Trizol Reagent (Invitrogen, Carlsbad) according to the manufacturer’s protocol followed by cDNA synthesis and PCR procedures. cDNA synthesis and PCR procedures were performed as described previously^62^. The PCR program was set at 94 °C for 5 min; 35 cycles of 94 °C for 45 s, 57 °C for 45 s, and 72 °C for 1 min; followed by 72 °C for 1 min. Relative expression levels were calculated using the 2^−ΔΔCT^ method^63^ and normalized to the reference GAPDH gene. The PCR products were further confirmed by sequencing (Sangon Biotechnology Co., Shanghai, China). The expressions of the following genes were detected: DSPP, DMP-1, OCN, OPN, Decorin, ALP, Periostin, COL-III, Fibronectin. Sequences of primers are showed in Supplementary Table S1.

### Western Blotting

At 3, 7, 14 days, Western Blotting was used to detect the differences in protein expression between JBMMSCs induced by TDM. Cells were collected by centrifugation, and protein extraction was performed using radio immunoprecipitation assay (RIPA) lysis buffer containing protease inhibitor cocktail (Sigma-Aldrich). Protein concentrations were determined using the bicinchoninic acid assay (Thermo Scientific, Waltham, MA). Proteins were then separated by 10% sodium dodecyl sulfate-polyacrylamide gel electrophoresis (SDS-PAGE; Bio-Rad, Hercules, CA)-based electrophoresis. The separated proteins were transferred onto a polyvinylidene fluoride (PVDF) membrane (Bio-Rad). The primary antibodies included COL-I (1:1000, abcam, USA), DMP-1 (1: 1000, Millipore, Germany), DSPP (1: 1000, Santa Cruz, USA), Periostin (1: 1000, Santa Cruz, USA), CAP (1: 1000, Santa Cruz, USA), Decorin (1: 1000, Santa Cruz, USA), RUNX2 (1: 1000, abcam, USA), OCN (1: 1000, abcam, USA), OPN (1: 1000, abcam, USA), GAPDH (1:5000, Abcam, USA). After several washes in Tris-buffered saline (Bio-Rad) with 0.2% Tween (America, Solon, OH), the membranes were incubated with the corresponding secondary antibody (goat anti-rabbit and goat anti-mouse) (1:5000, KeyGEN, China) and then developed using electrochemiluminescence (ECL) agents (Millipore, Darmstadt, Germany). To compare the relative protein intensity of each group, loading was normalized using GAPDH expression before analysis. Gray value of protein was measured by ImageJ software.

### Tissue regeneration *in vivo* using TDM and JBMMSCs

Twelve immunodeficient mice were divided into three groups including one test group (TDM combined with JBMMSCs) and one control group (single TDM). JBMMSCs were seeded onto TMD at a density of 1 × 10^4^ cells/scaffold and incubated at 37 °C for three days. Implantation of scaffolds was performed under deep anesthesia. Eight weeks later, all samples were obtained from the immunodeficient mice under deep anesthesia. Implants were fixed with 4% paraformaldehyde overnight at 4 °C, demineralized with 10% EDTA (pH 8.0), and embedded in paraffin. Paraffin sections were stained with hematoxylin and eosin stain (H&E), Mason stain and immunohistochemical stains.

Immunohistochemical antibodies included DMP-1 (1:200, Millipore, Germany), DSPP (1:200, Santa Cruz, USA), Decorin (1:200, Santa Cruz, USA), Periostin (1:200, Santa Cruz, USA), OCN (1:200, abcam, USA), OPN (1:200, abcam, USA), TGF-β (1:150, abcam, USA), Fironectin (1:200, abcam, USA), COL-I (1:200, abcam, USA), COL-III (1:500, abcam, USA), ALP (1:200, Zen, China), Mitochondria (1:200, Millipore, Germany). All antibodies were used according to the manufacturers’ protocol.

All animal experiments were conducted in accordance with the committee guidelines of Sichuan University for animal experiments, which also, meets the NIH guidelines for the care and use of laboratory animals. Immunodeficient mice were obtained from the Laboratory Animal Research Centre of Sichuan University and were maintained on a daily ration of Purina rodent chow in housing quarters with cycled light (12 h on/off), regulated temperature, and sterile water.

### Statistical analysis

All data were expressed as the mean ± SD. Statistical significance was analyzed using SPSS 16.0 software (SPSS, USA). A value of p < 0.05 was considered statistically significant.

## Additional Information

**How to cite this article:** Luo, S. *et al*. Bone marrow mesenchymal stem cells combine with Treated dentin matrix to build biological root. *Sci. Rep.* **7**, 44635; doi: 10.1038/srep44635 (2017).

**Publisher's note:** Springer Nature remains neutral with regard to jurisdictional claims in published maps and institutional affiliations.

## Supplementary Material

Supplementary Information

## References

[b1] Mao J. J. & Prockop D. J. Stem cells in the face: tooth regeneration and beyond. Cell Stem Cell 11, 291–301 (2012). 22958928 10.1016/j.stem.2012.08.010 PMC4093804

[b2] Fugazzotto P. A. Evidence-based decision making: replacement of the single missing tooth. Dent Clin North Am 53, 97–129, ix (2009). 19215747 10.1016/j.cden.2008.10.001

[b3] Li R. . Human treated dentin matrix as a natural scaffold for complete human dentin tissue regeneration. Biomaterials 32, 4525–4538 (2011). 21458067 10.1016/j.biomaterials.2011.03.008

[b4] Guo W. . Dental follicle cells and treated dentin matrix scaffold for tissue engineering the tooth root. Biomaterials 33, 1291–1302 (2012). 22088889 10.1016/j.biomaterials.2011.09.068

[b5] Yen A. H. & Sharpe P. T. Stem cells and tooth tissue engineering. Cell Tissue Res 331, 359–372 (2008). 17938970 10.1007/s00441-007-0467-6

[b6] Dannan A. Dental-derived Stem Cells and whole Tooth Regeneration: an Overview. J Clin Med Res 1, 63–71 (2009). 22505970 10.4021/jocmr2009.03.1230 PMC3318856

[b7] Li Z. Y. . Odontogenic potential of bone marrow mesenchymal stem cells. J Oral Maxillofac Surg 65, 494–500 (2007). 17307598 10.1016/j.joms.2006.09.018

[b8] Bianco P., Riminucci M., Gronthos S. & Robey P. G. Bone marrow stromal stem cells: nature, biology, and potential applications. Stem Cells 19, 180–192 (2001). 11359943 10.1634/stemcells.19-3-180

[b9] Orbay H., Tobita M. & Mizuno H. Mesenchymal stem cells isolated from adipose and other tissues: basic biological properties and clinical applications. Stem Cells Int 2012, 461718 (2012). 22666271 10.1155/2012/461718 PMC3361347

[b10] Seo B.-M. . Investigation of multipotent postnatal stem cells from human periodontal ligament. The Lancet 364, 149–155 (2004). 10.1016/S0140-6736(04)16627-0 15246727

[b11] Yu J. . Odontogenic capability: bone marrow stromal stem cells versus dental pulp stem cells. Biol Cell 99, 465–474 (2007). 17371295 10.1042/BC20070013

[b12] Ohazama A., Modino S. A., Miletich I. & Sharpe P. T. Stem-cell-based tissue engineering of murine teeth. J Dent Res 83, 518–522 (2004). 15218039 10.1177/154405910408300702

[b13] Nakashima M. & Akamine A. The application of tissue engineering to regeneration of pulp and dentin in endodontics. J Endod 31, 711–718 (2005). 16186748 10.1097/01.don.0000164138.49923.e5

[b14] Rodriguez-Lozano F. J. . Mesenchymal dental stem cells in regenerative dentistry. Med Oral Patol Oral Cir Bucal 17, e1062–1067 (2012). 22926467 10.4317/medoral.17925 PMC3505703

[b15] Zheng Y. . Dentin regeneration using deciduous pulp stem/progenitor cells. J Dent Res 91, 676–682 (2012). 22660968 10.1177/0022034512449834

[b16] Yuan Z. . Biomaterial selection for tooth regeneration. Tissue Eng Part B Rev 17, 373–388 (2011). 21699433 10.1089/ten.teb.2011.0041 PMC3179624

[b17] Chun S. Y. . Analysis of the soluble human tooth proteome and its ability to induce dentin/tooth regeneration. Tissue Eng Part A 17, 181–191 (2011). 20695775 10.1089/ten.TEA.2010.0121

[b18] Yang B. . Tooth root regeneration using dental follicle cell sheets in combination with a dentin matrix - based scaffold. Biomaterials 33, 2449–2461 (2012). 22192537 10.1016/j.biomaterials.2011.11.074

[b19] Shinohara Y., Tsuchiya S., Hatae K. & Honda M. J. Effect of vitronectin bound to insulin-like growth factor-I and insulin-like growth factor binding protein-3 on porcine enamel organ-derived epithelial cells. Int J Dent 2012, 386282 (2012). 22567008 10.1155/2012/386282 PMC3332072

[b20] Mitsiadis T. A., Graf D., Luder H., Gridley T. & Bluteau G. BMPs and FGFs target Notch signalling via jagged 2 to regulate tooth morphogenesis and cytodifferentiation. Development 137, 3025–3035 (2010). 20685737 10.1242/dev.049528 PMC3114538

[b21] Mammoto T. . Mechanochemical control of mesenchymal condensation and embryonic tooth organ formation. Dev Cell 21, 758–769 (2011). 21924961 10.1016/j.devcel.2011.07.006 PMC3199351

[b22] Okubo K. . Participation of endogenous IGF-I and TGF-beta 1 with enamel matrix derivative-stimulated cell growth in human periodontal ligament cells. J Periodontal Res 38, 1–9 (2003). 12558931 10.1034/j.1600-0765.2003.01607.x

[b23] Zhang W., Ahluwalia I. P. & Yelick P. C. Three dimensional dental epithelial-mesenchymal constructs of predetermined size and shape for tooth regeneration. Biomaterials 31, 7995–8003 (2010). 20682455 10.1016/j.biomaterials.2010.07.020 PMC3379891

[b24] Sonoyama W. . Mesenchymal stem cell-mediated functional tooth regeneration in swine. PLoS One 1, e79 (2006). 17183711 10.1371/journal.pone.0000079 PMC1762318

[b25] Scheller E. L., Krebsbach P. H. & Kohn D. H. Tissue engineering: state of the art in oral rehabilitation. J Oral Rehabil 36, 368–389 (2009). 19228277 10.1111/j.1365-2842.2009.01939.x PMC2744808

[b26] Honda M. J., Tsuchiya S., Sumita Y., Sagara H. & Ueda M. The sequential seeding of epithelial and mesenchymal cells for tissue-engineered tooth regeneration. Biomaterials 28, 680–689 (2007). 17045644 10.1016/j.biomaterials.2006.09.039

[b27] Liu J. . Matrix and TGF-beta-related gene expression during human dental pulp stem cell (DPSC) mineralization. In Vitro Cell Dev Biol Anim 43, 120–128 (2007). 17516126 10.1007/s11626-007-9022-8

[b28] Park E. S. . Proteomics analysis of human dentin reveals distinct protein expression profiles. J Proteome Res 8, 1338–1346 (2009). 19193101 10.1021/pr801065s

[b29] Holzwarth J. M. & Ma P. X. Biomimetic nanofibrous scaffolds for bone tissue engineering. Biomaterials 32, 9622–9629 (2011). 21944829 10.1016/j.biomaterials.2011.09.009 PMC3195926

[b30] Wallace J. M. . Type I collagen exists as a distribution of nanoscale morphologies in teeth, bones, and tendons. Langmuir 26, 7349–7354 (2010). 20121266 10.1021/la100006a PMC2868935

[b31] Weber I. T., Harrison R. W. & Iozzo R. V. Model structure of decorin and implications for collagen fibrillogenesis. J Biol Chem 271, 31767–31770 (1996). 8943211 10.1074/jbc.271.50.31767

[b32] Haruyama N. . Genetic evidence for key roles of decorin and biglycan in dentin mineralization. Matrix Biol 28, 129–136 (2009). 19379665 10.1016/j.matbio.2009.01.005 PMC2683192

[b33] Volk S. W. . Type III collagen regulates osteoblastogenesis and the quantity of trabecular bone. Calcified Tissue International 94, 621–631 (2014). 24626604 10.1007/s00223-014-9843-x PMC4335719

[b34] Piattelli A., Rubini C., Fioroni M., Tripodi D. & Strocchi R. Transforming growth factor-beta 1 (TGF-beta 1) expression in normal healthy pulps and in those with irreversible pulpitis. Int Endod J 37, 114–119 (2004). 14871177 10.1111/j.0143-2885.2004.00758.x

[b35] Horst O. V., Horst J. A., Samudrala R. & Dale B. A. Caries induced cytokine network in the odontoblast layer of human teeth. BMC Immunol 12, 9 (2011). 21261944 10.1186/1471-2172-12-9 PMC3036664

[b36] Nakashima M., Nagasawa H., Yamada Y. & Reddi A. H. Regulatory role of transforming growth factor-beta, bone morphogenetic protein-2, and protein-4 on gene expression of extracellular matrix proteins and differentiation of dental pulp cells. Dev Biol 162, 18–28 (1994). 8125185 10.1006/dbio.1994.1063

[b37] Toyono T., Nakashima M., Kuhara S. & Akamine A. Temporal changes in expression of transforming growth factor-beta superfamily members and their receptors during bovine preodontoblast differentiation *in vitro*. Arch Oral Biol 42, 481–488 (1997). 9296267 10.1016/s0003-9969(97)00041-1

[b38] Smith A. J. . Reactionary dentinogenesis. Int J Dev Biol 39, 273–280 (1995). 7626417

[b39] Edwards P. C. & Mason J. M. Gene-enhanced tissue engineering for dental hard tissue regeneration: (2) dentin-pulp and periodontal regeneration. Head Face Med 2, 16 (2006). 16725030 10.1186/1746-160X-2-16 PMC1481630

[b40] Baker S. M. . TGF-beta/extracellular matrix interactions in dentin matrix: a role in regulating sequestration and protection of bioactivity. Calcif Tissue Int 85, 66–74 (2009). 19424740 10.1007/s00223-009-9248-4

[b41] Lu Y. . Rescue of odontogenesis in Dmp1-deficient mice by targeted re-expression of DMP1 reveals roles for DMP1 in early odontogenesis and dentin apposition *in vivo*. Dev Biol 303, 191–201 (2007). 17196192 10.1016/j.ydbio.2006.11.001 PMC2059935

[b42] Beniash E. . Possible role of DMP1 in dentin mineralization. J Struct Biol 174, 100–106 (2011). 21081166 10.1016/j.jsb.2010.11.013 PMC3073716

[b43] Yamakoshi Y. Dentin Sialophophoprotein (DSPP) and Dentin. J Oral Biosci 50, 33–44 (2008). 20037676 10.2330/joralbiosci.50.33 PMC2797732

[b44] Reed C. C. & Iozzo R. V. The role of decorin in collagen fibrillogenesis and skin homeostasis. Glycoconj J 19, 249–255 (2002). 12975602 10.1023/A:1025383913444

[b45] Yamaguchi Y., Mann D. M. & Ruoslahti E. Negative regulation of transforming growth factor-beta by the proteoglycan decorin. Nature 346, 281–284 (1990). 2374594 10.1038/346281a0

[b46] Schonherr E., Broszat M., Brandan E., Bruckner P. & Kresse H. Decorin core protein fragment Leu155-Val260 interacts with TGF-beta but does not compete for decorin binding to type I collagen. Arch Biochem Biophys 355, 241–248 (1998). 9675033 10.1006/abbi.1998.0720

[b47] Ferdous Z., Wei V. M., Iozzo R., Hook M. & Grande-Allen K. J. Decorin-transforming growth factor- interaction regulates matrix organization and mechanical characteristics of three-dimensional collagen matrices. J Biol Chem 282, 35887–35898 (2007). 17942398 10.1074/jbc.M705180200

[b48] Sreenath T. . Dentin sialophosphoprotein knockout mouse teeth display widened predentin zone and develop defective dentin mineralization similar to human dentinogenesis imperfecta type III. J Biol Chem 278, 24874–24880 (2003). 12721295 10.1074/jbc.M303908200

[b49] Goldberg M. . Targeted disruption of two small leucine-rich proteoglycans, biglycan and decorin, excerpts divergent effects on enamel and dentin formation. Calcif Tissue Int 77, 297–310 (2005). 16283572 10.1007/s00223-005-0026-7

[b50] Sundberg M. . Expression of cementum-derived attachment protein in bovine tooth germ during cementogenesis. Bone 29, 242–248 (2001). 11557368 10.1016/s8756-3282(01)00573-7

[b51] Lian J. B. . Networks and hubs for the transcriptional control of osteoblastogenesis. Reviews in Endocrine & Metabolic Disorders 7, 1–16 (2006). 17051438 10.1007/s11154-006-9001-5

[b52] Sun H., Wu C., Dai K., Chang J. & Tang T. Proliferation and osteoblastic differentiation of human bone marrow-derived stromal cells on akermanite-bioactive ceramics. Biomaterials 27, 5651–5657 (2006). 16904740 10.1016/j.biomaterials.2006.07.027

[b53] Zhang W., Walboomers X. F., van Osch G. J., Van d. D. J. & Jansen J. A. Hard tissue formation in a porous HA/TCP ceramic scaffold loaded with stromal cells derived from dental pulp and bone marrow. Tissue Engineering Part A 14, 285–294 (2008). 18333781 10.1089/tea.2007.0146

[b54] Hunter G. K., Kyle C. L. & Goldberg H. A. Modulation of crystal formation by bone phosphoproteins: structural specificity of the osteopontin-mediated inhibition of hydroxyapatite formation. Biochemical Journal 300(Pt 3), 723–728 (1994). 8010953 10.1042/bj3000723 PMC1138226

[b55] Ma D. . A novel role of periostin in postnatal tooth formation and mineralization. J Biol Chem 286, 4302–4309 (2011). 21131362 10.1074/jbc.M110.140202 PMC3039381

[b56] Suzuki H. . Immunohistochemical localization of periostin in tooth and its surrounding tissues in mouse mandibles during development. Anatomical Record Part A Discoveries in Molecular Cellular & Evolutionary Biology 281A, 1264–1275 (2004). 10.1002/ar.a.20080 15386274

[b57] Tuli R. . Characterization of multipotential mesenchymal progenitor cells derived from human trabecular bone. Stem Cells 21, 681–693 (2003). 14595128 10.1634/stemcells.21-6-681

[b58] Song L., Young N. J., Webb N. E. & Tuan R. S. Origin and characterization of multipotential mesenchymal stem cells derived from adult human trabecular bone. Stem Cells Dev 14, 712–721 (2005). 16433626 10.1089/scd.2005.14.712

[b59] Shi S., Robey P. G. & Gronthos S. Comparison of human dental pulp and bone marrow stromal stem cells by cDNA microarray analysis. Bone 29, 532–539 (2001). 11728923 10.1016/s8756-3282(01)00612-3

[b60] Harada S. & Rodan G. A. Control of osteoblast function and regulation of bone mass. Nature 423, 349–355 (2003). 12748654 10.1038/nature01660

[b61] Chen G. . Combination of aligned PLGA/Gelatin electrospun sheets, native dental pulp extracellular matrix and treated dentin matrix as substrates for tooth root regeneration. Biomaterials 52, 56–70 (2015). 25818413 10.1016/j.biomaterials.2015.02.011

[b62] Wu G. . Odontogenic potential of mesenchymal cells from hair follicle dermal papilla. Stem Cells Dev 18, 583–589 (2009). 18673019 10.1089/scd.2008.0066

[b63] Livak K. J. & Schmittgen T. D. Analysis of relative gene expression data using real-time quantitative PCR and the 2(-Delta Delta C(T)) Method. Methods 25, 402–408 (2001). 11846609 10.1006/meth.2001.1262

