# Supplementary material for: Bone marrow mesenchymal stem cells combine with Treated dentin matrix to build biological root
Source: Sci Rep. 2017 Apr 12;7:44635. doi: 10.1038/srep44635 (PMC5388852; doi:10.1038/srep44635)
Supplement: Supplementary Information [file srep44635-s1.pdf]

Supplementary material

Title: Bone marrow mesenchymal stem cells combine with Treated dentin matrix to build biological root

Authors: Shiwei Luo[1], Fei Pei[1], Wen Zhang, Weihua Guo, Rui Li[*], Wei He[*], Weidong Tian[*]

Table.S1 Oligonucleotide Primer Sequences

Target cDNA	Primer sequence (5'–3')	Product Size(bp)	NCBI.no
DMP-1	F CTCGCACACACTCTCCCACTCAAA	180	NM_004407.3
	R TGGCTTTCCTCGCTCTGACTCTCT		
DSPP	F GCAGCAATAGCAGTGAGAGCAGTGA	104	NM_014208.3
	R GCTGCTGTCACTATCGCTGCTGTTA		
Decorin	F GTCATAGAACTGGGCACCAATC	140	NM_001920.4
	R GTAAGGGAAGGAGGAAGACCTTG		
Periostin	F CACTCTTTGCTCCCACCAATA	173	NM_001330517.1
	R ATTTCCTTCCAGCGTCTCAA		
ALP	F TAAGGACATCGCCTACCAGCTC	170	NM_000478.4
	R TCTTCCAGGTGTCAACGAGGT		
OCN	F CTCACACTCCTCGCCCTATTG	166	NM_199173.5
	R CTCCCAGCCATTGATACAGGTAG		
OPN	F CAGTTGTCCCCACAGTAGACAC	127	NM_001251830.1
	R GTGATGTCCTCGTCTGTAGCATC		
COL-III	F TGGAGGATGGTTGCACGAAA	73	NM_000090.3
	R ACAGCCTTGCGTGTTCGATA		
Fibronectin	F TCAGCTTCCTGGCACTTCTG	147	NM_212478.2
	R TCTTGTCCTACATTCGGCGG		
GAPDH	F CTTTGGTATCGTGGAAGGACTC	132	NM_002046.3
	R GTAGAGGCAGGGATGATGTTCT		

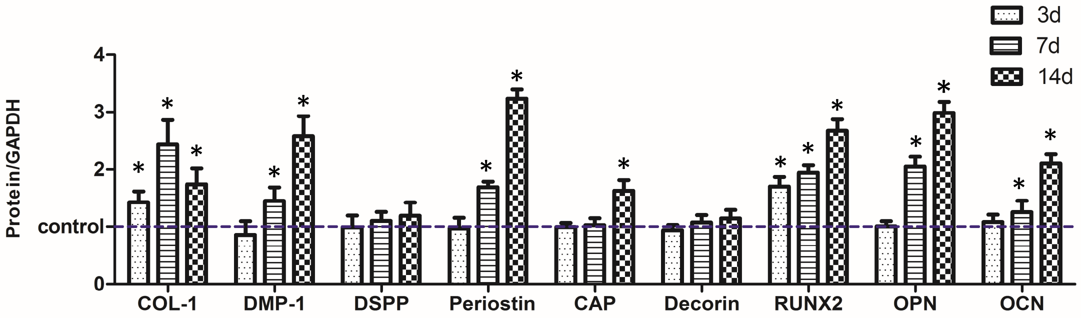

Fig.S1 Gray value statistical analysis of Western Blot. All proteins were levelled with GAPDH. And the ratios of all control group were adjusted to 1 which showed as a dotted line. (*, $p<0.05$)

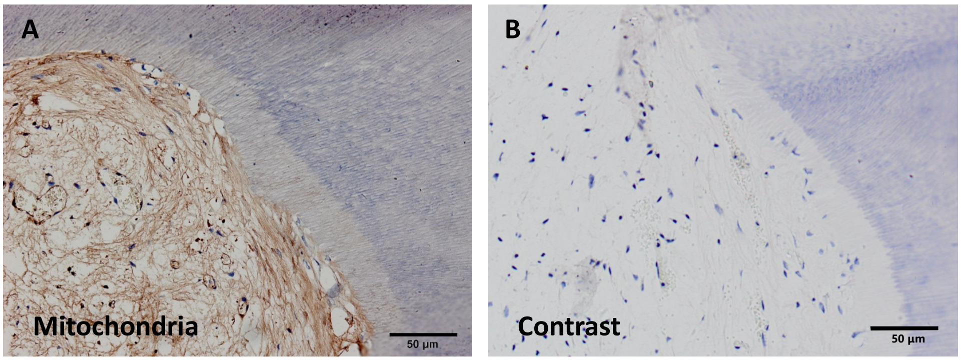

Fig.S2 Identify cells in harvest tissue with immunohistochemistry. (A)Cells were positively stained with human-Mitochondria. (B) Negative control (PBS replaced primary anti-body) didn’t show any positive expression.
